# Pitfalls in TAMVI: experience with the repositionable Lotus® Valve System

**DOI:** 10.1186/s13019-017-0615-3

**Published:** 2017-06-12

**Authors:** Paul P. Heinisch, Fabien Praz, Bernhard Winkler, Stephan Windecker, Christoph Huber, Thierry Carrel

**Affiliations:** 1 Department of Cardiovascular Surgery, Inselspital, University of Bern, Bern, Switzerland; 2 Department of Cardiology, Inselspital, University of Bern, Bern, Switzerland

**Keywords:** Transcatheter intervention, Left ventricular outflow tract obstruction, TAVI, TA-MVI, Case report

## Abstract

**Background:**

Simultaneous transapical implantation of transcatheter heart valves in the native mitral and aortic position may be considered as an alternative to surgical valve replacement in high-risk patients presenting with combined valve disease.

**Case presentation:**

A 59-year-old female with severe aortic stenosis, severe mitral stenosis with mild mitral insufficiency, persistent atrial fibrillation, severe chronic obstructive pulmonary disease and NYHA class of IV was evaluated by our interdisciplinary heart team. Due to the calculated Euroscore II, logistic Euroscore with 10% and 17% a decision was made towards a transapical TAVI approach. The implantation of a Sapien 3 (Edwards Lifesciences) valve in the aortic position was performed and the perioperative TEE showed a good result. The preoperative imaging revealed a narrow LVOT with risk for post interventional left ventricular outflow tract obstruction. Accordingly, it was decided against the use of balloon-expanding valves for the mitral valve position in the interdisciplinary team, as it is not repositionable. Instead, it was decided for the use of a Lotus (Boston Scientific) valve, as it is repositionable and therefore possible to retract in case of LVOT obstruction. In the present case of double valve intervention, the implantation attempt of a fully repositionable transcatheter heart valve into the native mitral annulus resulted in acute LVOT obstruction requiring immediate removal of the device. The patient was extubated and experienced uneventful postoperative recovery.

**Conclusions:**

The case shows that improved preoperative work-up is necessary for better prediction of significant LVOT obstruction following transcatheter mitral valve implantation. In borderline cases, the use of a fully repositionable device is preferred.

## Background

Transapical transcatheter aortic and mitral valve implantation (TA-AVI, TA-MVI) has become an attractive alternative to surgical valve replacement in high-risk patients presenting with significant aortic and mitral valve pathology [1]. However, only limited clinical experience is available so far and peri-procedural complication rate may be significant [2, 3].

Technical reasons may restrict the use of TAMVI, including optimal valve positioning, stable device anchoring precluding later valve migration [4], anatomical considerations such as the risk of left ventricular outflow tract (LVOT) obstruction and the occurrence of paravalvular leak in the D shaped mitral annulus [5]. For this purpose, the use of a repositionable device is a safe option to master the most important intra-procedural problems [6].

## Case presentation

**Fig. 1:**
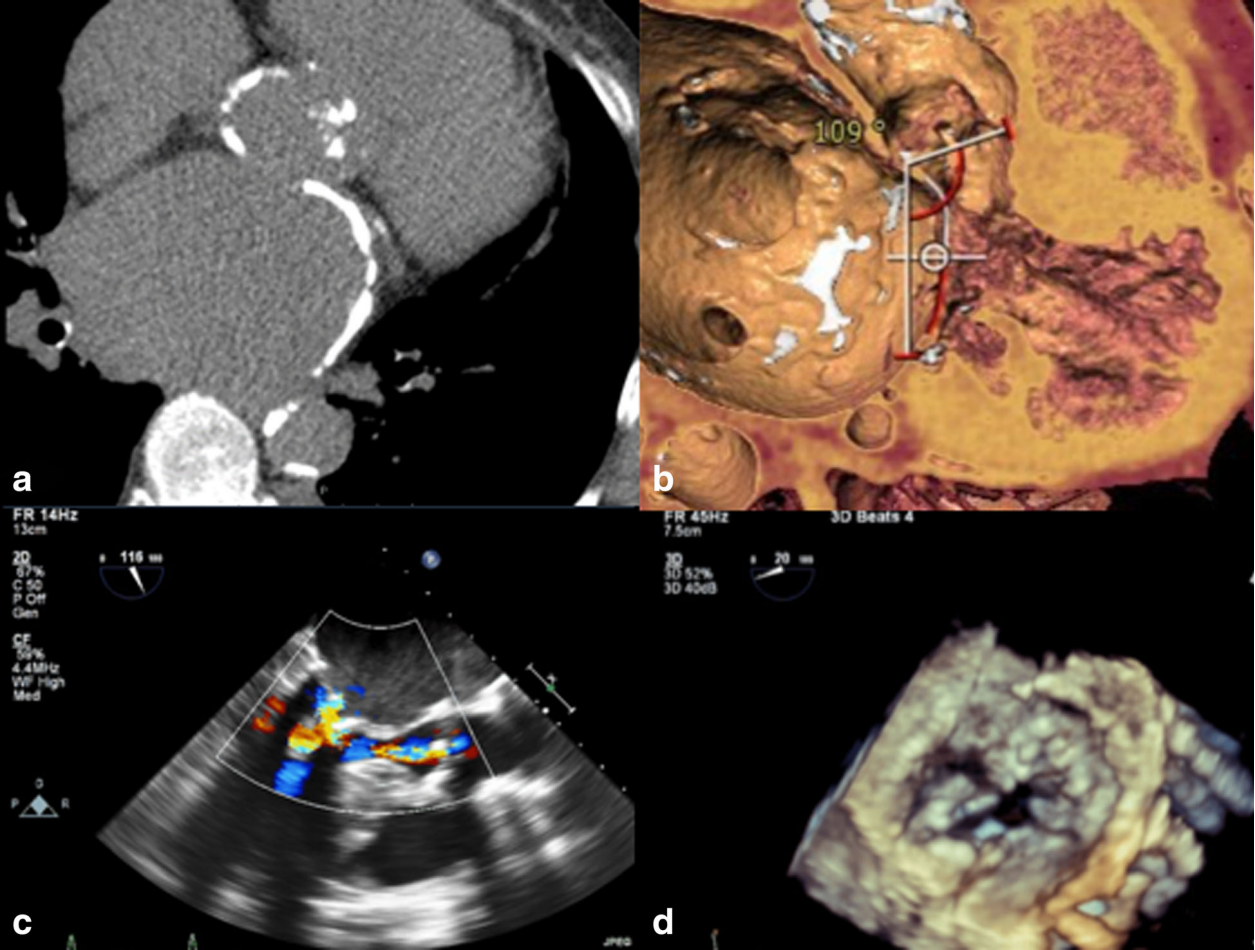
**a** Pre-operative CT-Scan: Severe calcification and dilation of *left atrium*. **b** CT-reconstruction with volume-rendering of the *left-ventricular* outflow tract: aorto-mitral angle of 109°. **c** TEE LVOT view in diastole, showing severe mitral valve stenosis and narrowing of the LVOT due to bulging of the inter-ventricular septum. **d** TEE 3D reconstruction of the native mitral valve during diastole showing severely decreased residual effective orifice area

A 59-year-old female highly symptomatic patient (functional class NYHA IV) with severe aortic and mitral valve stenosis combined to mild mitral regurgitation, was evaluated for a valve procedure by the institutional heart-team. She had undergone previous open mitral commissurotomy in 1979, and percutaneous mitral balloon valvuloplasty in 1989 and 2001. She suffered from persistent atrial fibrillation, severe chronic obstructive pulmonary disease, sleep apnea syndrome. Surgical valve replacement was deemed high risk, since she presented with a porcelain ascending aorta as well as massive calcifications of the left atrial wall (Fig. 1. a/b). Euroscore II and logistic Euroscore score were 10% and 17% respectively. Decision was made towards a transapical approach as a double-valve procedure. Preoperative imaging including angio-CT and transesophageal echocardiography (TEE) is demonstrated in Fig. 1. The mean transvalvular aortic gradient was 55 mmHg and the residual effective valve orifice area was 0.4 cm^2^. The preoperative left ventricular ejection fraction (LVEF) was measured at 55%. Furthermore, preoperative TEE revealed a LVOT diameter of 17 mm and a thickness of the subaortic septum at 14 mm. The aortic annulus perimeter 72.7 mm and perimeter-derived diameter 23.1 mm was measured using OsiriX MD DICOM viewer (© Pixmeo SARL) and 3Mensio (Pie Medical Imaging). The mitral valve showed a mean gradient of 12 mmHg with a residual valve orifice area of 0.57 cm^2^ calculated according to the pressure half-time method. Maximal diameter of the left ventricular outflow tract was 19 mm (Fig. 1.c). The aorto-mitral angle (AMA) was measured with 109° (Fig. 1 b) by 3Mensio (Pie Medical Imaging). The preoperative coronary angiogram revealed no signs of coronary artery disease. Furthermore, the examination revealed a moderate to severe post-capillary pulmonary hypertension with a mean of 44 mmHg.

**Fig. 2:**
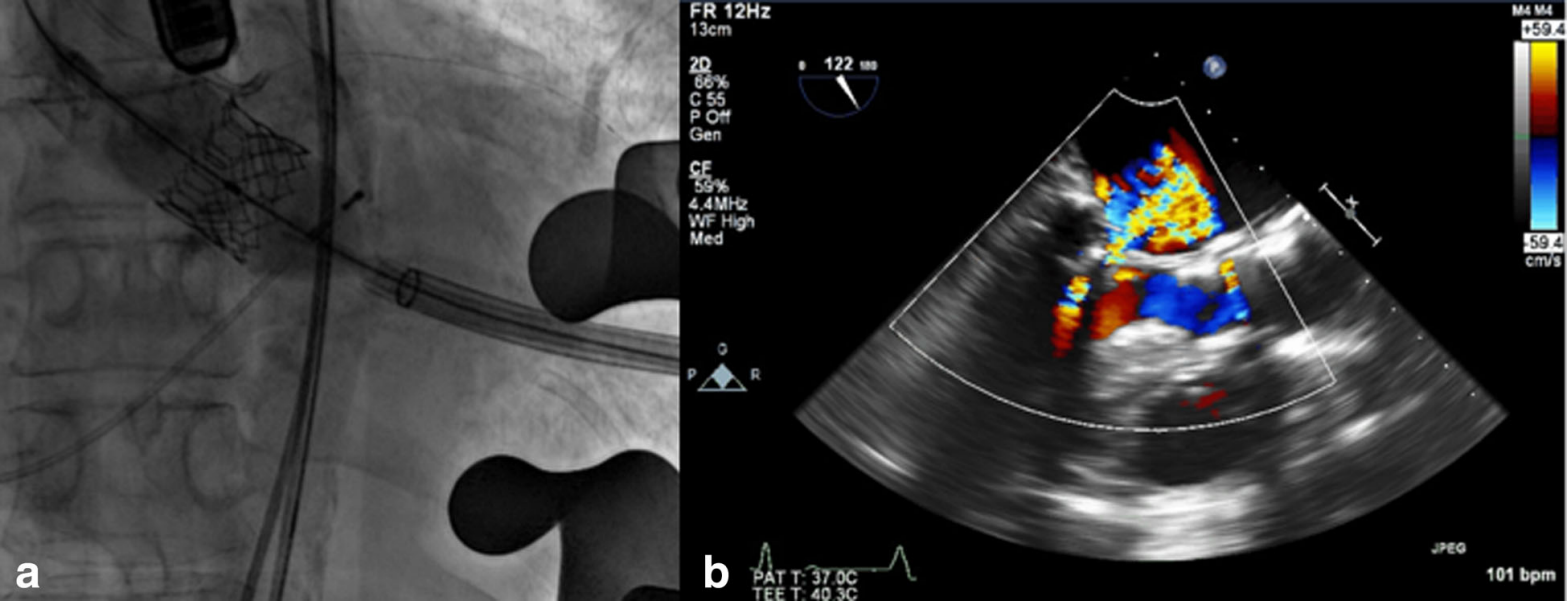
**a** Transapical deployment of the 23 mm Sapien 3 bioprosthesis in aortic position. **b** LVOT view showing minimal paravalvular regurgitation after successful implantation of an Edwards Sapien 3 23 mm valve in aortic position

The procedure was performed under general anesthesia utilizing both fluoroscopy and transesophageal echocardiography. A left mini-thoracotomy incision in the 5^th^ intercostal space, pre-dilatation of the aortic valve was performed with a 20 mm balloon and a 23 mm Sapien 3 valve (Edwards Lifesciences, Irvine, CA) was implanted under rapid ventricular pacing (Fig. 2a). The intraoperative TEE showed a very good aortic valve function without paravalvular regurgitation (Fig. 2b).

**Fig. 3:**
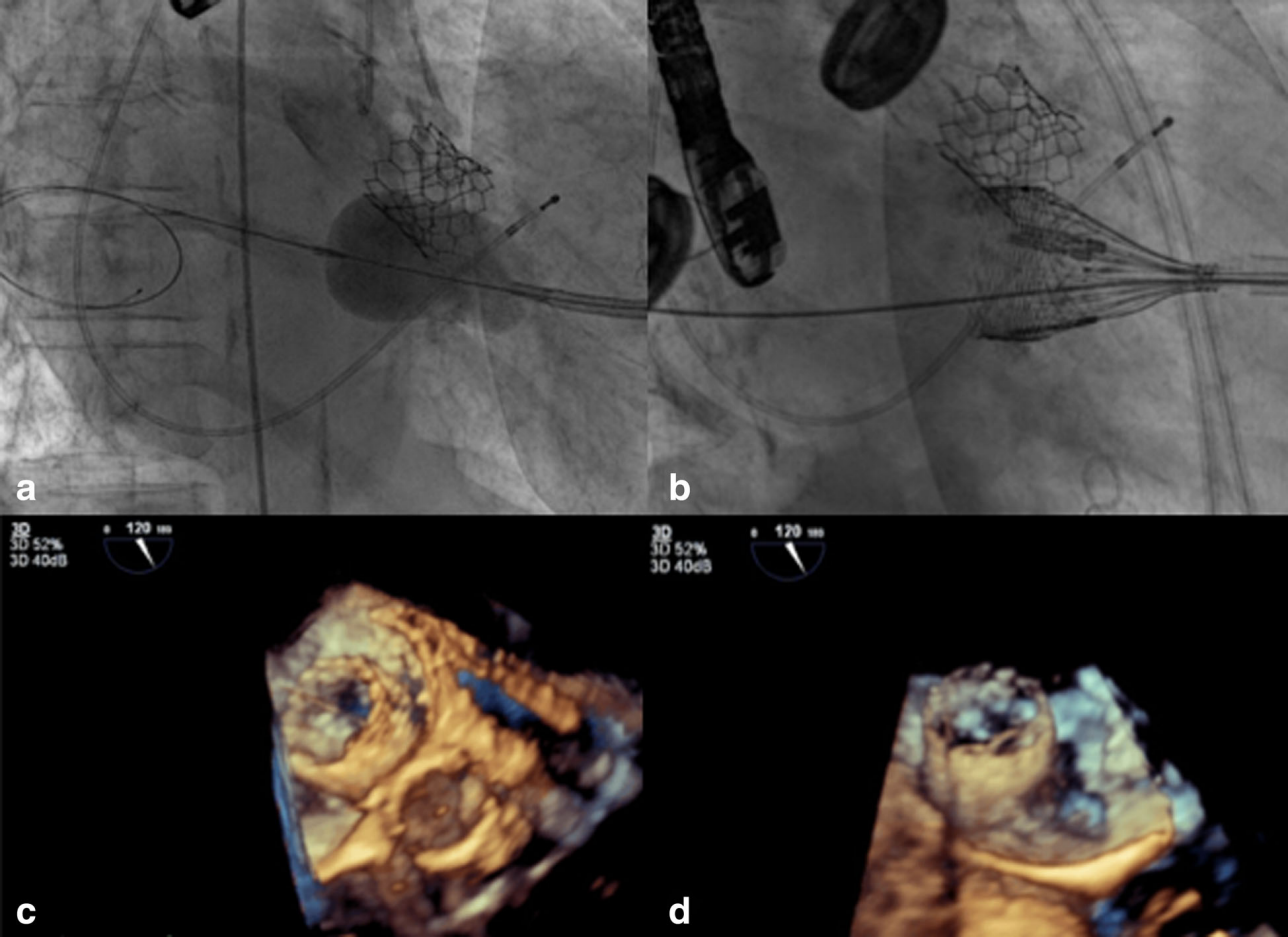
**a** Mitral valve pre-dilation with a 28 mm Inoue balloon inserted through the apex. **b** 27 mm Lotus Valve deployment in mitral position. **c** TEE 3D reconstruction of the atrial part of the Lotus Valve 27 mm in the native mitral annulus. **d** TEE 3D reconstruction of the atrial part of the Lotus Valve 27 mm in the native mitral annulus

Following the aortic valve procedure, repeated mitral balloon valvuloplasty was attempted using first a 28 mm Inoue balloon inserted through the apex and then a 30 mm Osypka balloon (Fig. 3a). The echocardiography showed a slight decrease of the mitral valve gradient to a mean value of 9 mmHg, but a moderate mitral valve regurgitation. Due to the insufficient dilatation of the mitral valve an attempt was undertaken to implant a 27 mm repositionable Lotus Valve System (Boston Scientific, Marlborough, MA) without sheath, directly through the exposed apex (Fig. 3b).

Immediately after the implantation of the Lotus valve, the patient developed profound hypotension. On echocardiography, severe obstruction of the left ventricular outflow tract (LVOT) due to anterior displacement of the thickened and partially calcified anterior native mitral valve leaflet was detected (Fig. 3c/d). The Lotus valve was partially recaptured and a second implantation was attempted with the valve positioned slightly higher into the left atrium, but LVOT-obstruction persisted. The valve was removed, leaving the native mitral valve with a transvalvular gradient of 4 mmHg, with mild mitral valve regurgitation.

The patient was extubated in the hybrid room and had an uneventful recovery. She was discharged to the rehabilitation clinic 9 days after the procedure. Echocardiography at one month showed mild aortic regurgitation and a moderate mitral valve stenosis with a mean gradient of 8 mmHg. Furthermore, the patient reported about an improvement of physical capacity after the intervention and rehabilitation.

## Discussion and conclusion

The development of transcatheter heart valves has led to profound changes in the treatment of valvular heart diseases. However, severe mitral valve stenosis is still challenging to treat percutaneously, as limited experience exists for transcatheter mitral valve implantation [7]. The calcification pattern commonly involves the valve leaflets, the annulus and the subvalvular apparatus. This represents a risk for LVOT obstruction [8]. The clinical experience with combined transapical transcatheter aortic and mitral valve replacement, is limited. In 2015 Akujuo reported a similar case [2]. The procedure requires careful planning, including precise morphological assessment with TEE and multi-slice CT. The procedure itself must be carefully guided by echocardiography and fluoroscopy. Especially the position of the mitral valved stent is crucial: if it is too low in the left ventricular cavity it may result in LVOT obstruction, if it is too high it increases the probability of thrombogenic complications in the left atrium [7, 8]. Morphology of the anterior mitral leaflet is important since following transcatheter placement, it can obstruct the LVOT. The success of the TA-MVI procedure depends on the correct deployment of the device, with the valve stent frame extending equally in the left ventricle and in the left atrium.

The obstruction of the LVOT is a critical complication after TAMVI. The anterior leaflet of the mitral valve can potentially obstruct the LVOT by the implanted transcatheter valve. Therefore, preoperative CT analysis is mandatory to identify risk factors for LVOT obstruction: left ventricular hypertrophy, increased septal thickness, aorto-mitral angle of <90° (AMA), small ventricular cavity and long anterior mitral leaflet. Other important factors are the selected valve and depth of implantation towards the left ventricle in relation to the mitral annulus [8, 9].

Our case emphasizes the importance of a most precise pre- and intra-procedural imaging to assess potential problems associated with the deployment of the transcatheter valves. Furthermore, the use of 3D models and printing for preoperative planning might help to reduce per-interventional complications and even improve outcome in high risk cases. In our patient, the LVOT was judged to be narrowed and the mitral valve morphology risky for LVOT obstruction. Accordingly, we decided against the use of the balloon-expandable Edwards Sapien 3 valve since, it is not repositionable and may have led to lethal LVOT obstruction. The decision to use the Lotus valve for this off-label indication was able to prevent irreversible hemodynamic compromise due to LVOT obstruction.

## Abbreviations

AMA: Aorto-mitral-angle
CT: Computed tomography
LVEF: Left ventricular ejection fraction
LVOT: Left ventricular outflow obstruction
TA-AVI: Transapical transcatheter valve aortic implantation
TA-MVI: Transapical transcatheter mitral valve implantation
TEE: Transesophageal echocardiography
